# Long lasting effects of early temperature exposure on the swimming performance and skeleton development of metamorphosing Gilthead seabream (*Sparus aurata* L.) larvae

**DOI:** 10.1038/s41598-021-88306-4

**Published:** 2021-04-22

**Authors:** Chara Kourkouta, Alice Printzi, George Geladakis, Nikos Mitrizakis, Nikos Papandroulakis, George Koumoundouros

**Affiliations:** 1 Biology Department, University of Crete, Vasilika Vouton, 70013 Heraklion, Crete Greece; 2 Institute of Aquaculture, Hellenic Centre for Marine Research, AquaLabs, 71500 Gournes, Heraklion, Greece

**Keywords:** Marine biology, Ichthyology

## Abstract

Temperatures experienced during early ontogeny significantly influence fish phenotypes, with clear consequences for the wild and reared stocks. We examined the effect of temperature (17, 20, or 23 °C) during the short embryonic and yolk-sac larval period, on the swimming performance and skeleton of metamorphosing Gilthead seabream larvae. In the following ontogenetic period, all fish were subjected to common temperature (20 °C). The critical swimming speed of metamorphosing larvae was significantly decreased from 9.7 ± 0.6 TL/s (total length per second) at 17 °C developmental temperature (DT) to 8.7 ± 0.6 and 8.8 ± 0.7 TL/s at 20 and 23 °C DT respectively (*p* < 0.05). Swimming performance was significantly correlated with fish body shape (*p* < 0.05). Compared with the rest groups, fish of 17 °C DT presented a slender body shape, longer caudal peduncle, terminal mouth and ventrally transposed pectoral fins. Moreover, DT significantly affected the relative depth of heart ventricle (VD/TL_,_ *p* < 0.05), which was comparatively increased at 17 °C DT. Finally, the incidence of caudal-fin abnormalities significantly decreased (*p* < 0.05) with the increase of DT. To our knowledge, this is the first evidence for the significant effect of DT during the short embryonic and yolk-sac larval period on the swimming performance of the later stages.

## Introduction

In 350 BC, ancient philosopher Aristotle observed that animals have different forms when developed in different environments^1^. Since then, the ability of a single genotype to produce different phenotypes in response to environmental conditions (phenotypic plasticity) has attracted research interest in a variety of plant and animal organisms^2–5^. Following the recent review of Burggren et al.^6^, it is nowadays suggested that *“genes, environment, development, epigenetic markers and stochastic changes in the developmental plan interact together”*, during critical ontogenetic periods, leading to phenotypic modifications resulting from developmental plasticity. The extent of the phenotypic response depends on the timing and width of the ontogenetic window challenged.

Global warming and seasonal temperature perturbations have increased our interest in the thermally induced phenotypic plasticity of poikilothermic organisms, including fishes. Within the tolerance zone, water temperature during the early life (developmental temperature) is an important driving factor of fish phenotypic plasticity, with consequences for the survival, growth and population structure of wild stocks^5,7,8^. The catalogue of plastic traits is extensive and includes traits like body shape^9,10^, meristic characters (e.g. number of fin-rays or vertebrae)^11^, sex^12,13^, muscle structure and enzyme activity^14,15^, thermal acclimation capacity^16^, ontogenetic scaling^17–19^, cardiac morphology^20^, lifespan^21^, stress and immune response^22^ or hypoxia tolerance^23^. With respect to the ontogenetic windows examined, research interest focuses on the embryonic or/and larval period, up to metamorphosis, because of the particular sensitivity of these stages to water temperature and the high magnitude of the resulting phenotypic responces^5^.

In the last decades, there is an increasing interest on the plastic responses of fish swimming speed and skeleton against water temperature. Swimming performance is one of the most important functional traits in developing fish, determining to a large extent the success of survival, dispersal and settlement^24,25^. To our knowledge, the scarce relevant literature demonstrates that water temperature during the embryonic and larval stages has a significant effect on the swimming performance of juveniles or adults (e.g., *Dicentrarchus labrax*^26^, *Danio rerio*^20^). The development of morphological defects in response to environmental factors is a kind of maladaptive phenotypic plasticity^5^. Literature abounds of studies on the effect of temperature on the development of early, and usually lethal morphological defects (e.g. notochord abnormalities, review by Boglione et al.^27^), whereas literature on the thermal optima for normal skeletal development is relatively limited^28–33^. Except of their negative effects on fish survival in nature, skeletal abnormalities are also a significant problem of product quality in reared fish^27^.

Gilthead seabream, *Sparus aurata* L., is a valuable species for fisheries and one of the most important species for European finfish aquaculture. It is a eurythermal species, with a spawning period extending for 3–4 months in the winter^34,35^ and a relatively wide optimum temperature range for normal development (16–24 °C)^31,36^. Existing literature suggests that Gilthead seabream is highly responsive to developmental temperature during the embryonic and larval stages (within a range of 16–22 °C), with significant modifications of the juvenile phenotype^22,31,37,38^. These modifications include fast myotomal muscle fibres^37^, skeletal abnormalities^31^, body shape^38^, stress and immune response^22^.

**Figure 1:**
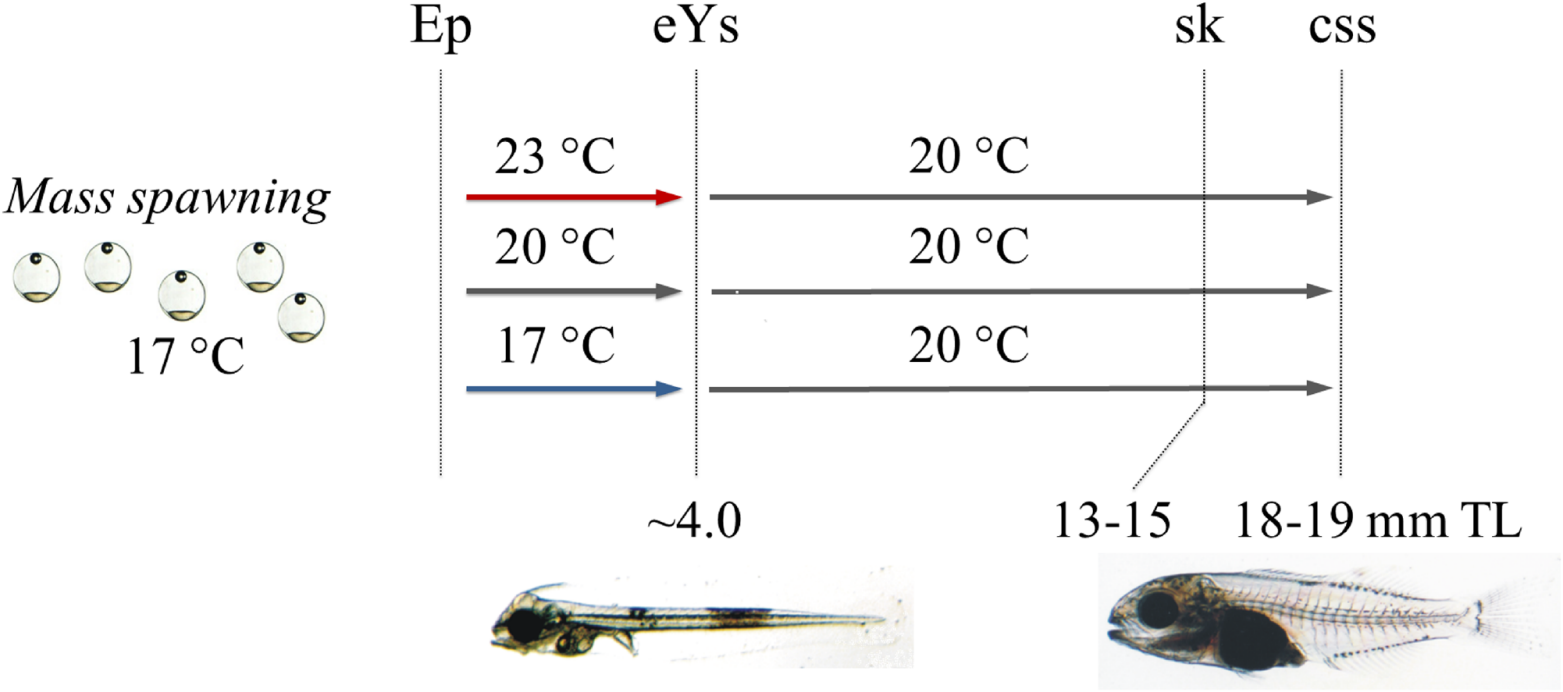
Experimental design. Fish were subjected to one of three developmental temperature treatments (17, 20, 23 °C) from the epiboly onset (Ep) to end of yolk-sac larval stage (eYs) and then to a common temperature up to the middle of metamorphosis (18–19 mm TL). All thermal treatments were applied in triplicate. css, critical swimming speed tests, body-shape and heart-shape analysis. sk, analysis of skeletal abnormalities.

In the present study we examined the effect of developmental temperature on the swimming performance of Gilthead seabream metamorphosing larvae. In contrary to previous relevant studies in other fish species, where temperature was applied during a relatively long ontogenetic period (embryonic and larval stages)^20,26^, in our study, the application of the different thermal treatments was limited to the short period of the embryonic and yolk-sac larval stages (Fig. 1). Body-shape and heart morphometry, important features for swimming speed^20,39^ were also analysed in an attempt to explain swimming performance results. Additionally, we examined the effect of temperature during this short ontogenetic period on the development of skeletal abnormalities.

## Results

### Swimming performance

**Figure 2:**
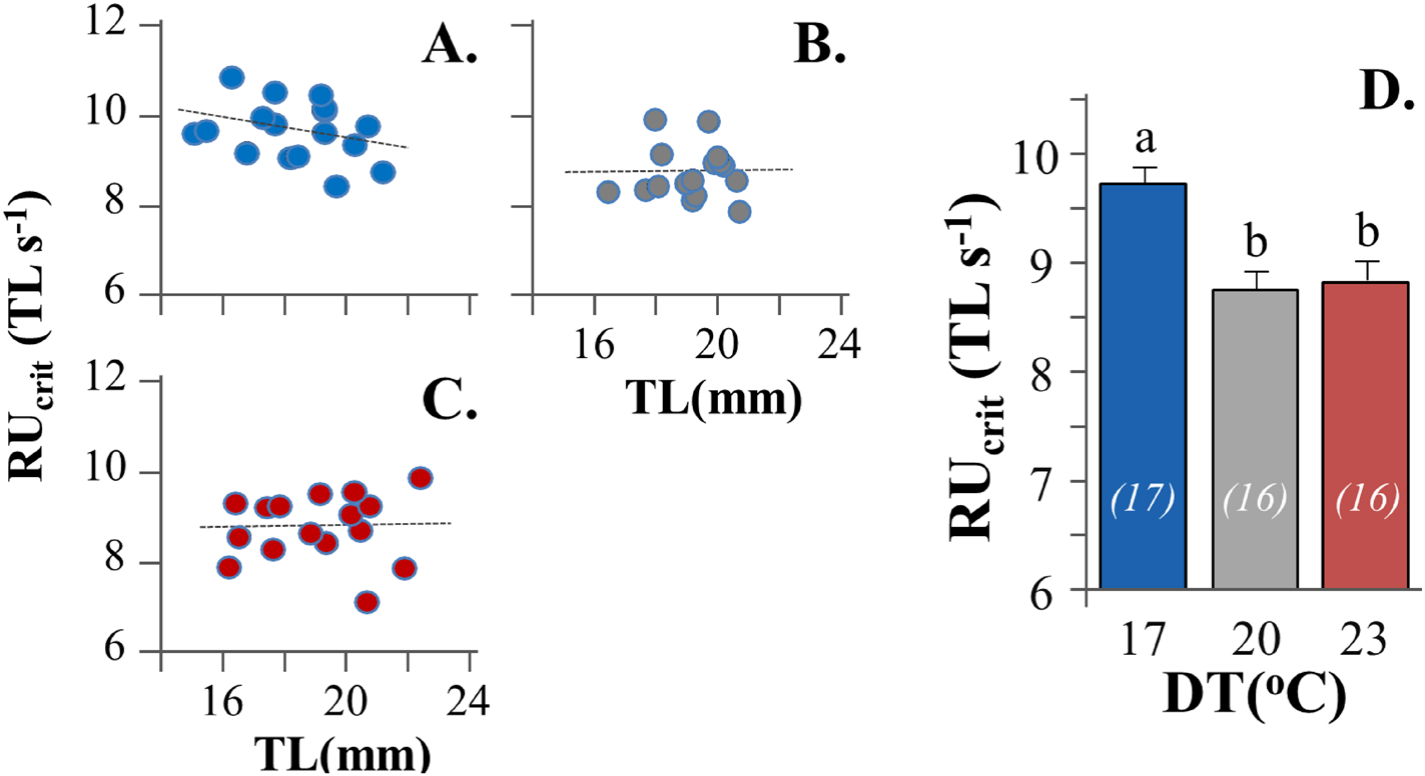
Effect of developmental temperature on the relative critical swimming speed (RU_crit_) of seabream metamorphosing larvae. (**A**–**C**) RU_crit_-TL graph for 17, 20 and 23 °C developmental temperature (DT) respectively. (**D**) Mean RU_crit_ in the different DTs. Values without a letter in common are statistically different (*p* < 0.05, Kruskal–Wallis and Mann–Whitney U test). Error bars equal to 1 SE. Numbers in parentheses indicate the sample size.

In all experimental conditions tested, relative critical swimming speed (RU_crit_) was independent of fish total length (TL, *p* > 0.05, Fig. 2A–C). Developmental temperature (DT) had a significant effect on the critical swimming speed of seabream metamorphosing larvae (*p* < 0.05). Fish initially reared at 17 °C DT achieved significantly higher swimming speed (9.7 ± 0.6 TL s^-1^, mean ± SD) than those reared at 20 (8.7 ± 0.6 TL s^-1^) and 23 °C DT (8.8 ± 0.7 TL s^-1^) (Fig. 2D). No significant differences were present in the TL of exercised fish between the different experimental groups (*p* > 0.05, Table S2).

### Body-shape and heart morphometry

**Figure 3:**
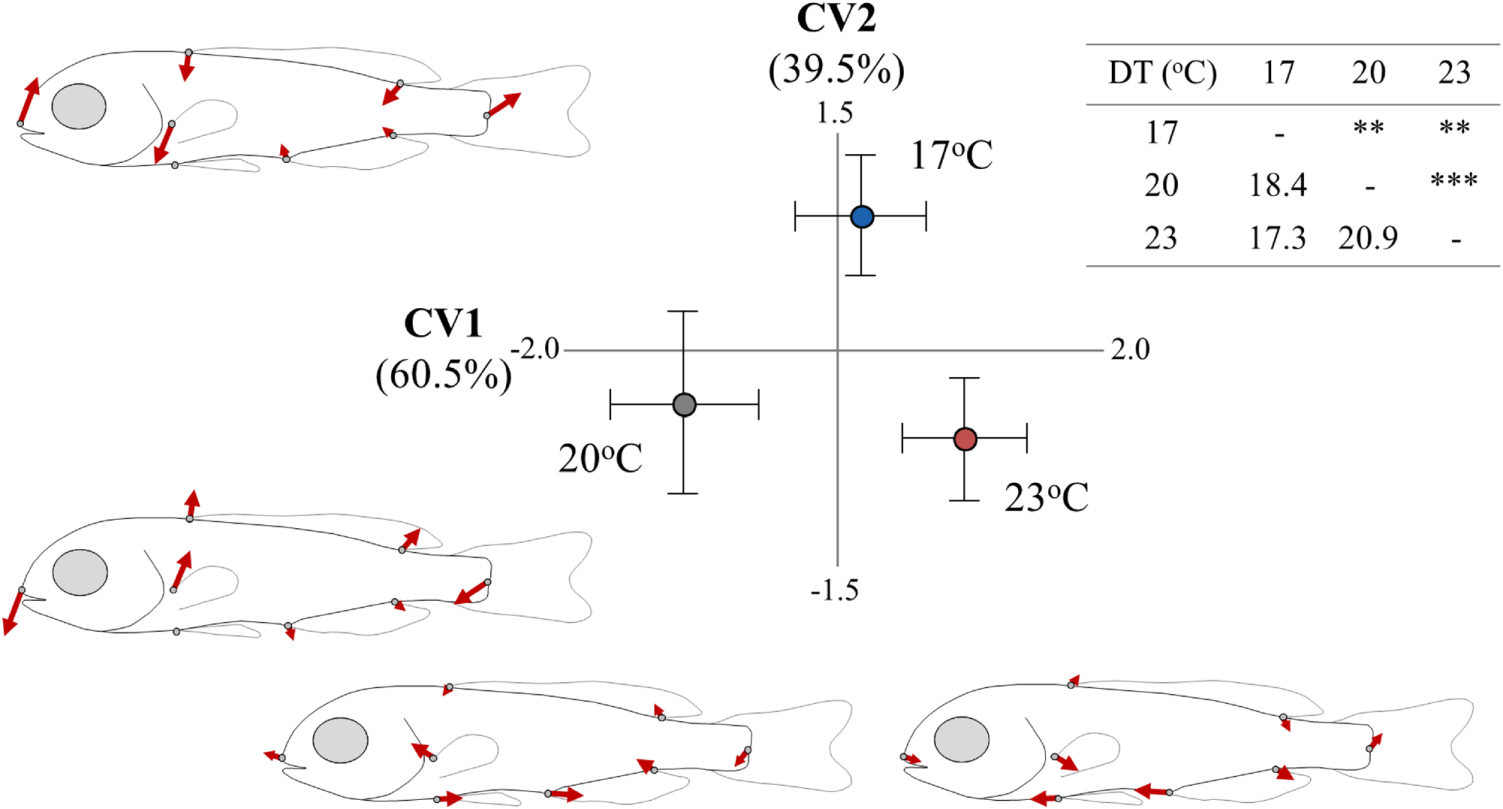
Effect of developmental temperature (17, 20, 23 °C) on the body-shape of seabream metamorphosing larvae. Means (± 2SE) of the canonical scores are given. Numbers in brackets are equal to the percentage of shape variance explained by each canonical variate (CV) axis. Vector diagrams demonstrate the components of shape change relative to the extreme values of CV axes. Squared Mahalanobis distances between the different groups and the respective significance levels are given in the table next to CVA graph. **p < 0.01; ***p < 0.001.

Developmental temperature had a significant effect on fish body shape (*p* < 0.05, Table S3), with significant squared Mahalanobis distances between all the experimental groups (Fig. 3). The second canonical variate (CV2, 39.5% explained variance) discriminated the fish of 17 °C DT from the rest two experimental groups (Fig. 3), and was significantly correlated with RU_crit_ (Pearson *r* = 0.36, *p* < 0.05). Compared with the rest two groups, the fish of 17 °C DT were characterised by a dorso-ventrally compressed body-shape (ventral shift of the dorsal fin), longer caudal peduncle, ventrally shifted pectoral fins and terminal position of the snout (Fig. 3). No significant correlation was observed between CV1 (first canonical variate) and fish swimming speed (Pearson *r* = − 0.03, *p* > 0.05).

**Figure 4:**
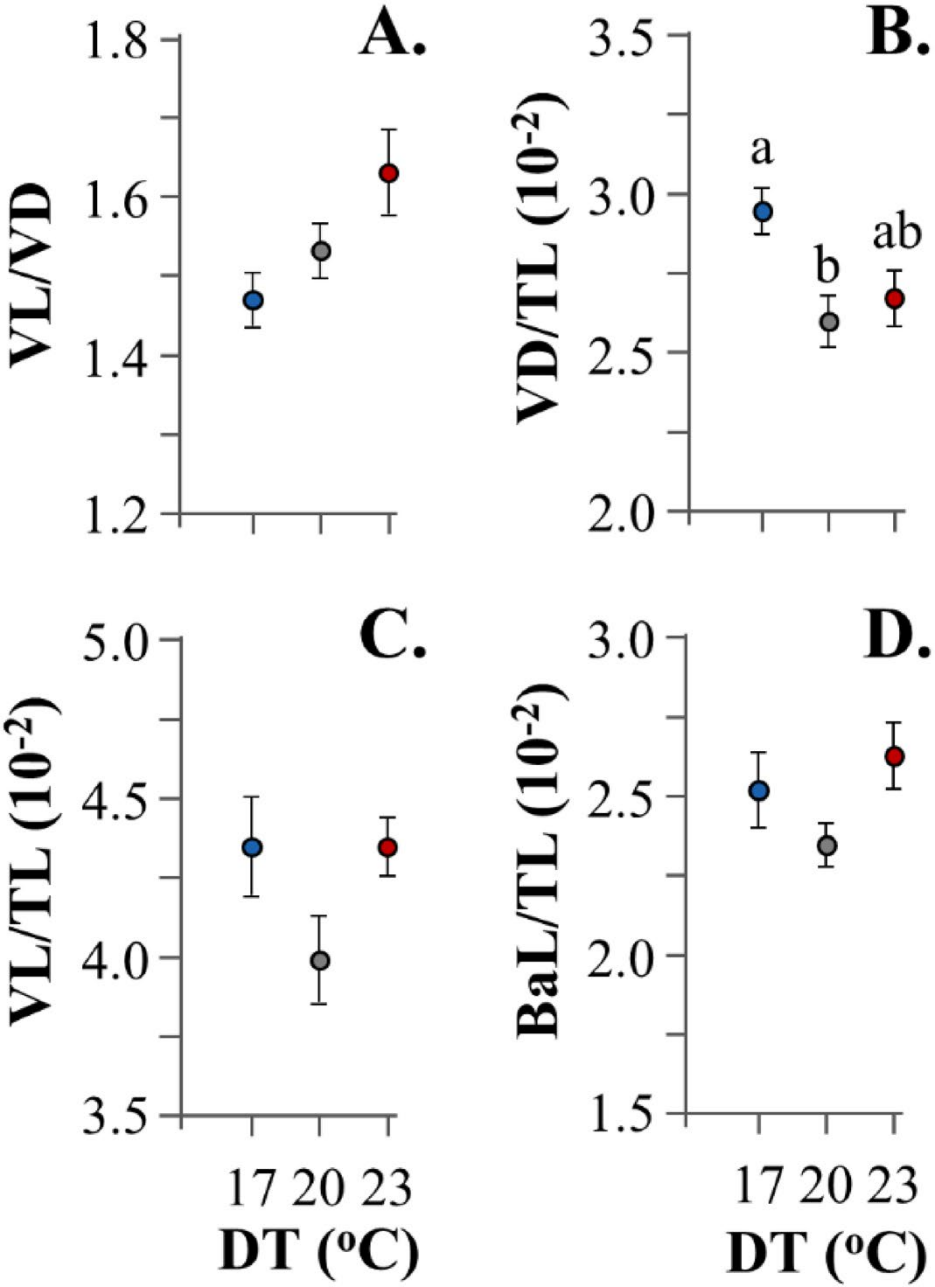
Effect of developmental temperature (DT) on the cardiac anatomy of seabream metamorphosing larvae. BaL, bulbus-arteriosus length. TL, total length. VD, ventricle depth. VL, ventricle length. Values without a letter in common are statistically different (*p* < 0.05, Kruskal–Wallis and Mann–Whitney U test). Data represent mean values (± 1 SE).

Heart morphometric analysis revealed no significant temperature effects on ventricle shape (ventricle length-to-depth ratio, VL/VD, *p* > 0.05, Fig. 4A). Developmental temperature significantly affected the relative ventricle depth (VD/TL_,_ *p* < 0.05), which was elevated at the lower temperature tested (Fig. 4B). Rest cardiac size variables (VL, BaL) were not significantly affected by DT (Fig. 4C,D).

### Skeletal deformities

**Figure 5:**
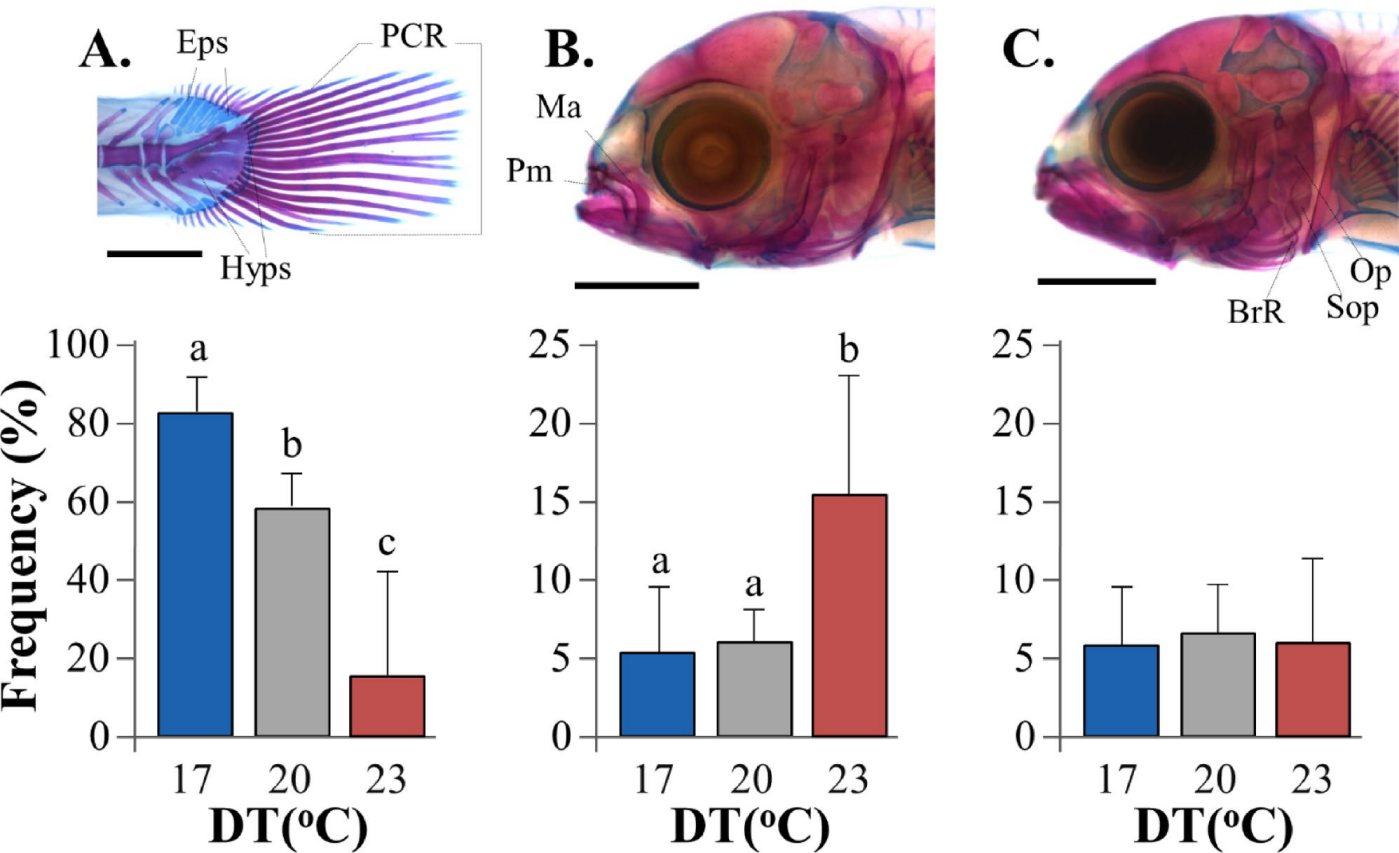
Effect of developmental temperature (DT) on the frequency of skeletal abnormalities in seabream larvae. (**A**) Caudal-fin abnormalities. (**B**) Pugheadness. (**C**) Gill-cover abnormalities. Eps, epurals. Hyps, hypurals. PCR, principal caudal-fin rays. Ma, maxillary. Pm, pre-maxillary. Op, operculum. Sop, sub-operculum. BrR, branchiostegal rays. Values without a letter in common are statistically different (*p* < 0.05, G-test). Data represent mean values. Error bars equal to 1 SD. Alcian Blue, Alizarin Red S staining.

Caudal-fin, upper jaw and gill-cover were the anatomical areas with the most frequent and severe skeletal abnormalities at 13–15 mm TL (Fig. 5). Caudal-fin abnormalities consisted of lack of ca. 1–4 lepidotrichia and fin structure, and were associated with severe abnormalities of caudal-fin supporting elements. Their frequency was significantly decreased as developmental temperature rose from 17 (82.7 ± 9.0%) to 23 °C (16.0 ± 26.0%, *p* < 0.05, Fig. 5A). Upper jaw abnormalities consisted of pugheadness and were associated with severe abnormalities of the maxillary and pre-maxillary bones. Their incidence was significantly elevated as DT increased from 17–20 °C (5.3 ± 4.2–6.0 ± 2.0%) to 23 °C (15.3 ± 7.6%, *p* < 0.05, Fig. 5B). No significant effects of DT were observed in the case of gill-cover abnormalities (6.0–6.3%), which consisted of inside folding of the operculum, sub-operculum and branchiostegal rays (*p* > 0.05, Fig. 5C). Miscellaneous abnormalities of very low rates (0.7 ± 1.2 to 4.0 ± 2.0% in total) and no significant differences between experimental regimes consisted of crossbite, lordosis or saddleback syndrome.

A remarkable intra-group variability was present in 23 °C DT, with one replicate presenting significantly higher abnormality rates than the other two (Fig. S2).

### Growth and survival rate

Survival rate was 36 ± 5%, 36 ± 2% and 27 ± 6% (± SD) for the 17, 20 and 23 °C DT group respectively, without any significant differences between the experimental groups (*p* > 0.05, Kruskal–Wallis test). The growth curve of all the examined populations presented a clear inflexion point at 31 dph, that divided the studied period into a phase of faster (< 35 dph) and a phase of slower growth rate (Fig. S1). For both growth phases, no significant differences were observed in the growth rate between the different thermal regimes (ANCOVA, *p* > 0.05, Table S4).

## Discussion

### Critical swimming speed

In the present paper, we found that water temperature during early development influences the swimming performance of metamorphosing seabream larvae. To our knowledge, this is the first study demonstrating the programming of swimming performance by the temperature which was experienced by the fish during the relatively short embryonic and yolk-sac period. Previous relevant studies on the thermally induced programming of fish swimming performance, targeted the wider ontogenetic period from the embryonic stage to metamorphosis, in European sea bass^26^ and zebrafish^20^. Interestingly, and independent of the tested species and ontogenetic stage, all studies concluded that low developmental temperature results in higher fish swimming performance in the following developmental stages (present and previous^20,26^ studies).

Body shape is tightly associated with the swimming performance of fishes^40^. In the present paper, fish with the highest critical swimming speed (17 °C DT group) were characterized by a comparatively slender body, terminal mouth and ventrally transposed pectoral fins. Similar responses to low developmental temperature have been reported in European seabass metamorphosing larvae^41^ and in Gilthead seabream juveniles^38^. A streamline body form serves in reducing drag during prolonged swimming, whereas a lower orientation of the pectoral fins serves in improving hovering and manoeuvrability^42–44^. Whether the observed body shape plasticity of metamorphosing seabream larvae (present study) is reversible or not during the following development, remains unknown. In European seabass, thermally induced plasticity of body shape decreased during the metamorphosis period^41^. In Gilthead seabream, Loizides et al.^38^ showed that the plastic responses of juvenile body shape decreased as different thermal treatments were applied during shorter and earlier ontogenetic windows.

The plastic response of larval swimming performance to developmental temperature (present paper) might be also related to thermally-induced modifications in other traits than body-shape, which are known to affect swimming speed (e.g., properties of swimming muscles, energy metabolism, mitochondria number, cardiac shape)^16,20,39,45^. In the present paper, we showed that developmental temperature significantly affected the relative size of ventricle depth (VD/TL), but not the ventricle shape (VL/VD). Whether these changes have a significant effect on the observed differences in fish swimming speed remains unknown. Previous studies demonstrated that critical swimming speed is positively correlated with elongated heart ventricles (high VL/VD ratio) in rainbow trout^46^ and zebrafish^20,47^. In the former species, this correlation was attributed to the bigger maximum cardiac output of the less rounded ventricles^46^. Differences in the rates of skeletal abnormalities between the different thermal groups (present study) are unlikely to have a significant contribution to the observed plasticity in swimming performance, because only individuals without gross external malformations were tested for swimming speed.

The successful settlement of fish larvae to coastal areas depends on hydrodynamics, as well as on larval behaviour and swimming capabilities^48,49^. In the present study, swimming performance was tested on the basis of critical swimming speed, which is widely used to assess the environmental effects on fish aerobic performance^20,24,50–52^. It is considered as a convenient and relatively accurate experimental method to estimate sustained swimming speed of fish, which is primarily driven by aerobic, slow, red muscle fibres^53,54^. In spite of the recent criticism raised on the use of U_crit_ values in larval dispersal modelling^25^, critical swimming speed has been commonly used in the study of larval dispersal capabilities^55,56^. In the present paper, critical swimming speed was tested in metamorphosing larvae of 18–19 mm TL. At this stage seabream larvae settle to coastal areas^57,58^, where they will shift to the juvenile morphology (i.e. deeper body profile), muscle structure (i.e. relatively fewer red muscle fibres) and swimming mode (i.e. lower U_crit_)^39^. Following our results, high water temperatures during a short but early ontogenetic period (embryonic and yolk-sac larval stage) can substantially decrease the swimming performance (10% decrease in U_crit_) of metamorphosing seabream larvae, and thus potentially lower their probability to settle. In a recent study involving six coastal, temperate Mediterranean fish species and larval dispersal modelling, Faillettaz et al.^49^ suggested that the settlement rate of fish larvae is directly proportional to their critical swimming speed.

### Skeletal abnormalities

The development of skeletal abnormalities in reared fish has been attributed to the action of a great variety of causative environmental or genetic factors^27,59,60^. It is considered as a complex and often multiparametric process, where the optima for normal ontogeny may significantly change during fish ontogeny^61^, or may be altered by the action of other environmental parameters and genotype (cofactors)^27^. In the present paper we found that the water temperature during the embryonic and yolk-sac larval stage significantly affected the development of caudal fin and upper jaws in the following ontogenetic period. At the end of the yolk-sac larval stage many fish species, including seabream, are characterized by low differentiation rate, without any element of the caudal-fin formed and only few cranial elements at the beginning of their ontogeny (including the maxillaries)^62–64^. Although the formation of the first caudal-fin elements starts well after the end of the autotrophic phase (ca 5.6 mm TL in seabream), caudal-fin abnormalities have been shown to be associated with posterior notochord distortions which appear during the earlier ontogenetic period^62,65^. To our knowledge, no studies exist on the ontogeny of upper-jaw abnormalities.

In the present study, caudal fin abnormalities were significantly elevated at the lower temperatures tested (on average 83% at 17 °C, 58% at 20 °C), whereas upper-jaw abnormalities were elevated at the higher temperature (23 °C, 15% on average). In agreement with our results, Georgakopoulou et al.^31^ showed that the rate of caudal-fin abnormalities in reared seabream increases when early life stages are exposed to comparatively low temperature (i.e. 16 °C vs. 19–22 °C). The fact that the mean rate and severity degree of caudal-fin abnormalities were substantially lower in Georgakopoulou et al.^31^ than in the present study could be explained by differences in the genetic origin of the fish and possible genotype to environmental interactions (as in the case of growth traits)^66^. In contrary to our results, no significant effect of water temperature on the rate of jaw abnormalities was reported by Georgakopoulou et al.^31^. However, our findings on jaw abnormalities should be critically considered, since the between group differences were mainly due to only one out of the three experimental replicates at 23 °C (Fig. S2).

The mechanism how temperature controls the development of morphological abnormalities is still largely unknown^27,67^. A possible mechanism might be associated with thermally-induced alterations of molecular pathways which are involved in bone and notochord development (in the case of caudal-fin abnormalities). In general, water temperature might also influence bone development indirectly, via modifications of the species’ nutritional optima, or via changes of the nutritional status of the planktonic organisms used for the larval feeding^27,67^. In spite of the significant role of nutrition for the skeleton development^61,68,69^, in the present study, nutritional condition was not considered as an aspect, because different thermal treatments were applied before larvae start exogenous feeding. In the present study, applied temperature acclimation rate (0.2–0.5 °C h^−1^) is considered low^31,36^ and, thus unlikely to have induced the reported abnormalities. This is furthermore supported by the comparatively low abnormality rates in the treatment with the higher temperature changes (23 °C DT, Fig. 1). More research is required to understand how early thermal manipulations are linked with the development of caudal-fin abnormalities in the following larval period.

In the present study, optimum developmental temperature (DT) for normal skeletal development (23 °C) was different from that for swimming performance (17 °C). Similar differences in the optimum DT have been shown in European sea bass, in respect to the critical swimming speed^26^ and the normal development of the branchiostegal rays^30^, but not of the vertebral column^28^. Such differences in optimum DT levels between different traits might be attributed to trait-specific ontogenetic timing and sensitivity to temperature.

### Conclusions

Global warming has increased our interest in the effects of developmental temperature (DT) on fish phenotype, with existing studies focusing on different ontogenetic windows and a great variety of traits^5,70^. Our results show that elevations in water temperature during an early and short ontogenetic period (embryonic and yolk-sac larval) can have adverse effects on the swimming performance of metamorphosing Gilthead seabream larvae, with potential consequences for the settlement success of the wild seabream stocks. Despite the clear decrease of caudal-fin abnormalities with the elevation of DT, the significance of these results for seabream natural stocks remains under question, mainly because tested temperatures have been proven as optimal for normal seabream embryonic and yolk-sac larval development (16–24 °C)^36^. In the case of seabream hatcheries, our results suggest that thermal manipulations of fish embryos and larvae can potentially be associated with the development of skeletal abnormalities and the resulting decrease of product quality.

## Materials and methods

### Experimental design and fish maintenance

Nine groups of fish were subjected in triplicate to 17, 20 or 23 °C, from the stage of epiboly onset to the end of yolk-sac larval stage and the beginning of exogenous feeding (5–7 days post-fertilization, dpf). Subsequently, all groups were kept under identical rearing conditions and rearing temperature (20 ^ο^C) up to the end of the trial (18–19 mm TL, Fig. 1, Table S1). Acclimation of the eggs from the spawning temperature (17 °C) to the test temperatures, and then to the common temperature for larval rearing (20 °C) was performed at the rate of 0.5 and 0.2 °C h^−1^, respectively. All eggs came from the same mass, spontaneous spawn of captive breeders. In all the replicates, initial stocking density was 115 eggs L^-1^.

Egg hatching and larval rearing were performed by using the methodology of Papandroulakis et al.^71^. For the rearing, recirculation systems with 500 L, black, cylindro-conical tanks (one tank per replicate) and biological filters were used. Water temperature was adjusted to the test levels using chillers and heaters, which were automatically controlled with electronic sensors (Eliwell, EU). Larvae were successively fed on enriched (DHA Protein Selco, INVE) rotifers until they reached 6 mm total length (TL), enriched rotifers and *Artemia* nauplii (Easy DHA Selco, INVE) when they were between 6 and 10 mm TL and enriched Artemia nauplii after 10 mm TL. Rotifers concentration in the tanks was adjusted two times daily to 6–8 ind ml^−1^. The concentration of *Artemia* nauplii was adjusted twice daily to 0.5–2 ind ml^−1^. During the rotifer-feeding period, live microalgae *Chlorella* sp. were added three times daily at a concentration of 6.5 ± 3 × 10^5^ cells ml^−1^. Weaning to inert diets (O-range, INVE) started when fish were approximately 8 mm TL. During the rotifer-feeding period, the excess of rotifers was removed from the culture medium by means of planktonic traps (50 μm mesh size), positioned at the water-outlet of tanks. During the first twenty days of feeding period, air-blowing skimmers were installed to keep the water surface free from lipids and enhance swim bladder inflation. Water oxygen saturation was 5.8 ± 1.0 to 6.0 ± 0.9 mg/L^-1^, pH 7.8 ± 0.3 (Table S1), salinity 35–36 ‰ and photoperiod 18L:6D. Water turnover rate ranged from 20% of the tank volume d^-1^ during the autotrophic phase to 70% h^-1^ at the end of larval rearing. Water was pumped from a deep (> 100 m) borehole. Larval rearing was performed at the Institute of Marine Biology, Biotechnology and Aquaculture (HCMR).

### Swimming performance

Swimming performance was assessed by estimating the relative critical swimming speed (RU_crit_) at the middle of metamorphosis (Fig. 1, Table S2). Incremental swimming tests were performed according to Koumoundouros et al.^39^, with a custom-designed apparatus with a swimming channel of 70 cm length, 10 cm depth and 5 cm width. Flow regime was adjusted by means of external pumps and valves. An electromagnetic flow-meter (Valeport, model 801) was used to calibrate water speed in the tunnel. A screen of plastic straws helped in maintaining laminar flow in the swimming channel and in preventing fish forward escape. Temperature was maintained constant at 20 °C, oxygen saturation at 100% and salinity at 35 ‰.

Eighteen to twenty hours prior to the tests, ten fish from each experimental population were transferred to one holding aquarium and deprived of food. For the swimming trials, fish were placed in the swimming channel for 10 min at 2 TL s^−1^ water speed. In the following period, water velocity increased at a rate of 1 TL s^−1^ every 10 min. Swimming tests were terminated when fish were fatigued and left the swimming channel, unable to react to visual and acoustic stimuli from behind or the side^39^. Critical swimming speed (U_crit_) was calculated according to the formula U_crit_ = U_i_ + (U_ii_·t_i_/t_ii_), where U_i_ is the highest swimming velocity (mm s^−1^) maintained for a full interval of 10 min, U_ii_ the velocity increment (1 TL s^−1^), t_i_ is the time interval at the fatigue velocity, and t_ii_ is the time interval between water velocity changes (i.e. 10 min)^72^. Fatigued fish were anaesthetized (MS222, 100 mg/L^−1^), photographed under a stereoscope (Olympus SZ61), measured for ΤL (Image analysis, Lumenera Infinity Analyze Microscopy Software, version 6.5.4, Canada), fixed in 5% phosphate buffered formalin, and examined for the presence of gross morphological abnormalities. Only fish with a normal morphology were included in the analysis. Relative critical swimming speed (RU_crit_), was calculated as the ratio of U_crit_ to the TL of each individual. To test the independence of RU_crit_ on TL, we used a linear regression analysis and Student statistic for the significance of slope equality to zero. Kruskal–Wallis and Mann–Whitney U statistics were applied to test the effect of exercise temperature on RU_crit_ (α = 0.05) (IBM SPSS Statistics, IBM Corp. in Armonk, NY). In total, 16–17 individuals from two replicates (8–9 fish per experimental replicate) of each experimental condition were tested. Non-parametric tests were used because the assumptions of ANOVA test (normality, homoscedasticity) were not met^73^.

### Body shape analysis

**Figure 6:**
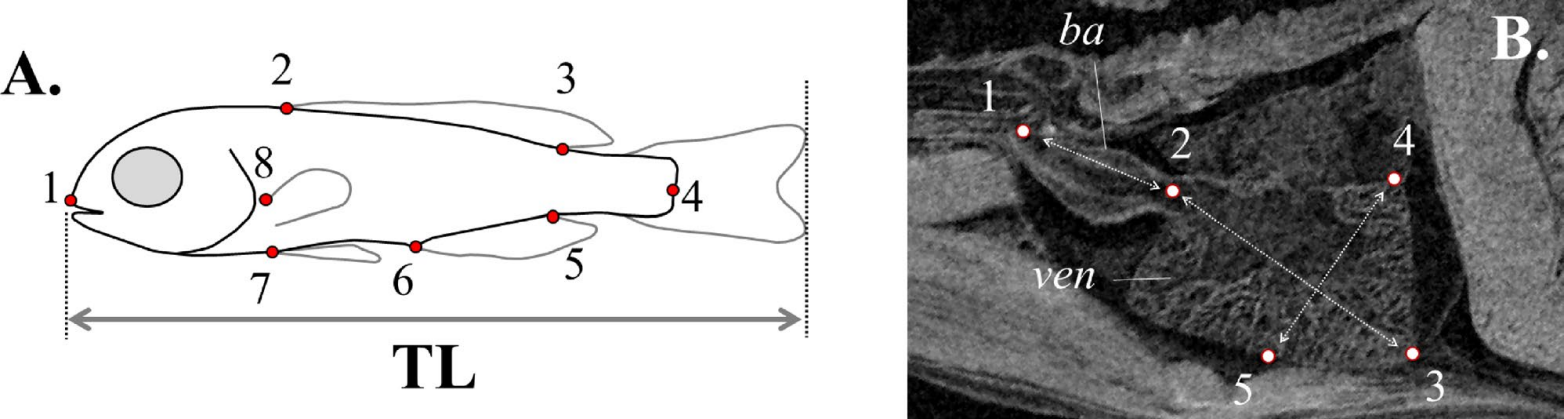
(**A**) Landmark and distance characters used in body-shape analysis. 1, Anterior tip of snout; 2 and 3, anterior and posterior base of the dorsal fin; 4, base of the central caudal lepidotrichium; 5 and 6, posterior and anterior base of the anal fin; 7, base of the pelvic fins; 8, dorsal base of the left pectoral fin; TL, total length. (**B**) Landmark and distance characters used in heart morphometric analysis. ba, bulbus arteriosus; ven, ventricle; 1, bulbus junction with the first branchial arch; 2, ventricle–bulbus valve; 3, ventricle apex; 4 and 5 landmarks define the widest ventricle depth, perpendicularly to ventricle length (D2–3); D1–2, Bulbus-arteriosus length (BaL); D2–3, Ventricle length (VL); D4–5, maximum ventricle depth (VD), perpendicular to VL.

Following swimming performance tests, eight landmark measurements were taken on the digital photographs of each fish (tpsDig2 software^74^, Fig. 6A). To extract shape information from the landmark configurations of body shape, the MorphoJ software^75^ was used. Shape information was extracted from the landmark data with Procrustes superimposition, by aligning the specimens by the principal axes of the mean shape configuration^76^. New superimposed landmark configurations were used to quantify the significance of the effect of developmental temperature on shape variation by Procrustes ANOVA^77^. To test and visualize the separation of body-shape among the different groups (thermal regimes) in multivariate space, a canonical variate analysis (CVA) was performed. *P*-values for the pair-wise differences between groups were estimated based on Mahalanobis distances, using 10,000 permutations per test^78^. To illustrate shape changes between thermal groups across the CV axes (CV1, CV2), vector diagrams ("lollipop graphs") were acquired^75^. The length and direction of each line in every graph indicates the movement of the respective landmark from the mean shape to a target shape, which corresponds to the minimum and/or the maximum value on each axis.

### Heart morphometry

Heart morphometric analysis was performed by micro-ct imaging, according to Dimitriadi et al.^20^. Following swimming tests, formalin fixed specimens were stained for six days with 2.5% PMA (phosphomolybdic acid hydrate, Sigma Aldrich), a contrast agent for soft tissue discrimination, and then gradually dehydrated in 70% ethanol. Stained fish were then individually scanned with a micro-ct scanner (SkyScan 1172, 2.3–3.3 μm resolution, 180° total rotation, 650 ms exposure time, 49–59 kV voltage, 167–200 μA). To avoid shrinkage from dehydration during scanning, specimens were kept in ethanol-saturated vials. The projection images which were obtained during the scanning process were reconstructed (NRecon software, SkyScan) into cross-sections and stored as TIFF-image stacks. The TIFF virtual images were then imported in the Amira v.5.2 software (Visage Imaging, Berlin, Germany, and Burlington, USA) to obtain a two- and three-dimensional representation of the cross-sectional image data. Morphometrics were taken on the sagittal plane which was defined by (a) the anterior end of bulbus arteriosus, (b) the distal tip of cleithrum bones, and (c) the centre of the 1st vertebra. The xyz coordinates of five distinct landmarks of the ventricle and the bulbus arteriosus, were retrieved by means of Amira. Landmarks were located on the base of the posterior aortic arch (landmark 1), the ventriculo-bulbar valve (landmark 2), the apex (landmark 3), distally to the widest distance of the ventricle, perpendicularly to ventricle length (landmarks 4 and 5) (Fig. 6B).

The effect of developmental temperature on heart morphometric indices was tested by means of the non-parametric Kruskal–Wallis and Mann–Whitney tests (α = 0.05) (IBM SPSS Statistics, IBM Corp. in Armonk, NY). In total, seven to eight individuals per experimental condition (3–4 per experimental replicate) were examined for heart morphometry. Non-parametric tests were used because the assumptions of ANOVA test (normality, homoscedasticity) were not met^73^.

### Skeletal deformities analysis

A random sample of 50 fish was taken from each experimental population at 13–15 mm TL, i.e. when most abnormalities have completed their development^67^ (Fig. 1). Fish were anaesthetized (ethylenglycol-monophenylether, 0.2–0.5 ml L^-1^) and fixed in phosphate buffered 5% formalin. All samples were stained for bone and cartilage^79^ and examined for the presence of skeletal abnormalities, following the terminology of Koumoundouros^67^. The study focused on severe abnormality types, excluding possible light malformations of single skeletal elements. The significance of the differences in the frequency of skeletal abnormalities among the different treatments was tested by means of G-test (α = 0.05)^73^.

### Growth and survival

A random sample of 10 larvae was taken every 2–7 days from each experimental population. Larvae were anesthetized (ethylenglycol-monophenylether, 0.2–0.5 ml L^-1^) and their total length was measured under the stereoscope (Fig. 6A). Specific growth rates were estimated by fitting age-TL data to the logarithmic model

$$TL = a \times e^{SGR \times t} ,$$

where α is the intercept (mm), SGR is the specific growth rate (day^-1^) and t is the age (days post-hatching, dph)^80^. The differences in the SGR among the different populations or between different growth phases of the same population were tested by using ANCOVA (α = 0.05)^73^. Fish survival rate was estimated at the end of the larval rearing phase (61 dph), after counting survived fish in all the experimental populations. The effect of temperature on fish survival rate was tested by means of the non-parametric Kruskal–Wallis and Mann–Whitney tests (α = 0.05). Non-parametric tests were used because the assumptions of ANOVA test (normality, homoscedasticity) were not met^73^.

### Ethical statement

All methods were carried out in accordance with relevant guidelines and regulation. All methods are reported in accordance with ARRIVE guidelines. HCMR installations at Crete are Licensed Facilities for operations of breeding & experimental use of fish issued by Region of Crete, General Directorate of Agricultural & Veterinary No 3989/01.03.2017 (approval codes EL91-BIObr-03 and EL91-BIOexp-04). The experimental protocol has been approved by the Veterinarian Authority of the Region of Crete with the 255332/29-11-2017 document.

## Supplementary Information

Supplementary Information.

## Data Availability

The data that support the findings of this study are available from the corresponding author upon reasonable request.
