# Supplementary material for: Long lasting effects of early temperature exposure on the swimming performance and skeleton development of metamorphosing Gilthead seabream (Sparus aurata L.) larvae
Source: Sci Rep. 2021 Apr 22;11:8787. doi: 10.1038/s41598-021-88306-4 (PMC8062446; doi:10.1038/s41598-021-88306-4)
Supplement: Supplementary file 1 — Supplementary Information. [file 41598_2021_88306_MOESM1_ESM.docx]

**Long lasting effects of early temperature exposure on the swimming performance and skeleton development of metamorphosing Gilthead seabream (*Sparus aurata* L.) larvae**

Chara Kourkouta^1^, Alice Printzi^1^, George Geladakis^1^, Nikos Mitrizakis^2^, Nikos Papandroulakis^2^, George Koumoundouros^1*^

^1^ Biology Department, University of Crete, Vasilika Vouton, 70013 Heraklion, Crete, Greece, tel. +30 2810394065, Fax. +30-2810394408, [gkoumound@uoc.gr](mailto:gkoumound@uoc.gr)

^2^ Institute of Aquaculture, Hellenic Centre for Marine Research, AquaLabs, 71500, Gournes, Heraklion, Greece

*, to whom correspondence should be addressed

**Table S1.** Mean values (±SD) of temperature, oxygen concentration and pH in the different replicates (a, b, c) and thermal treatments. DT, water temperature during the embryonic and yolk-sac larval stages. T_com_, water temperature which was applied after the end of yolk-sac larval stage.

| Group | Rep | DT (^o^C) | T_com_ (^o^C) | O_2_ (mg L^-1^) | pH |
| --- | --- | --- | --- | --- | --- |
|  | a | 17.1±0.1 | 19.9±0.2 | 6.0±0.9 | 7.8±0.3 |
| 17 | b | 17.0±0.1 | 19.9±0.2 | 5.8±1.0 | 7.8±0.3 |
|  | c | 17.1±0.1 | 19.8±0.2 | 6.0±0.9 | 7.8±0.2 |
|  | a | 20.1±0.1 | 19.9±0.2 | 5.8±0.7 | 7.8±0.3 |
| 20 | b | 20.1±0.1 | 19.9±0.2 | 5.8±0.7 | 7.8±0.2 |
|  | c | 20.1±0.1 | 19.9±0.2 | 5.9±0.7 | 7.8±0.2 |
|  | a | 22.8±0.1 | 19.9±0.3 | 6.0±0.6 | 7.8±0.2 |
| 23 | b | 22.7±0.1 | 19.9±0.2 | 6.0±0.8 | 7.8±0.2 |
|  | c | 22.7±0.1 | 19.9±0.3 | 5.9±0.7 | 7.8±0.2 |

**Table S2.** Mean, SD and range of the total length (TL) of the fish which were tested for critical swimming speed. DT, developmental temperature. No significant between-group differences in TL (Kruskal-Wallis test, p>0.05)

| DT (^o^C) | mean TL (mm) | SD | Range (mm) | n |
| --- | --- | --- | --- | --- |
| 17 | 18.4 | 1.8 | 15.1-21.2 | 17 |
| 20 | 19.1 | 1.2 | 16.4-20.6 | 16 |
| 23 | 19.1 | 1.9 | 16.2-22.3 | 16 |

**Table S3**: Results of Procrustes ANOVA for the effect of developmental temperature on the body-shape of fish.

| Effect | SS | df | MS | *F* | *p* |
| --- | --- | --- | --- | --- | --- |
| Individual | 0.002 | 24 | 0.000 | 1.170 | <0.05 |
| Residual | 0.035 | 552 | 0.000 |  |  |

SS, sum of squares; df, degrees of freedom; MS, mean squares; *F*, *F* statistic;

**Table S4**: Parameters of the TL growth curve, during the period following the yolk-sac larval stage, at the three different thermal regimes and growth phases (<32 or >31 dph). DT, developmental temperature. SGR, specific growth rate. a, intercept. SE, standard error of the estimate. r^2^, coefficient of determination. n, number of samples in each regression analysis. Estimates with different superscript were significantly different (p<0.05, ANCOVA).

| Phase | DT (^o^C) | SGR | SE_SGR_ | a | SE_a_ | r^2^ | n |
| --- | --- | --- | --- | --- | --- | --- | --- |
| <32 dph | 17 | 0.041^a^ | 0.001 | 1.180^a^ | 0.017 | 99.0 | 24 |
|  | 20 | 0.043^a^ | 0.001 | 1.231^b^ | 0.015 | 99.1 | 26 |
|  | 23 | 0.042^a^ | 0.001 | 1.217^c^ | 0.015 | 99.0 | 27 |
| >31dph | 17 | 0.021^b^ | 0.001 | 1.844^d^ | 0.045 | 95.4 | 24 |
|  | 20 | 0.021^b^ | 0.002 | 1.890^e^ | 0.052 | 94.1 | 24 |
|  | 23 | 0.021^b^ | 0.001 | 1.907^e^ | 0.037 | 96.9 | 24 |


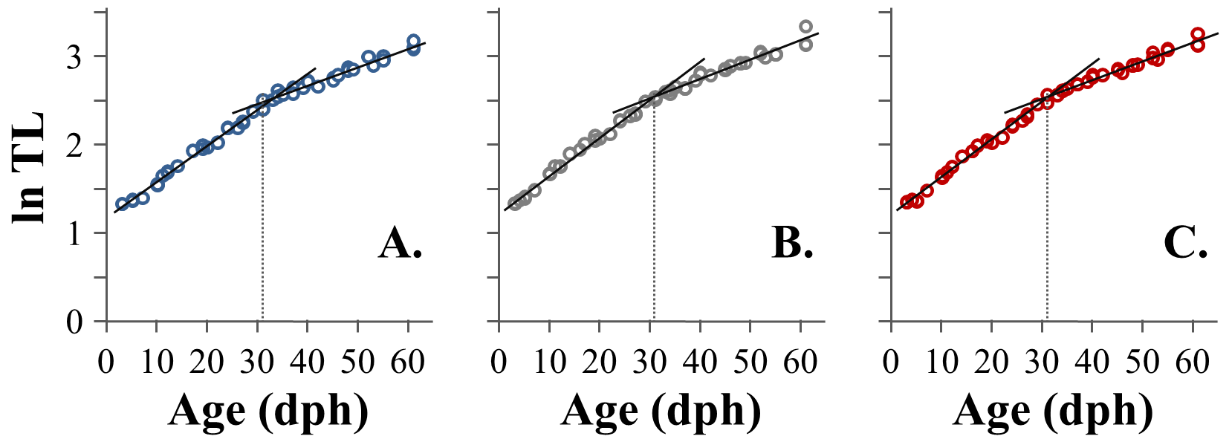


**Fig. S1**: Growth of fish total length (TL) with the age in the different thermal treatments. A clear inflection point in fish growth was observed at 31 dph (days post-hatching). Estimated growth rates and further statistical analyses are given in Table S4.


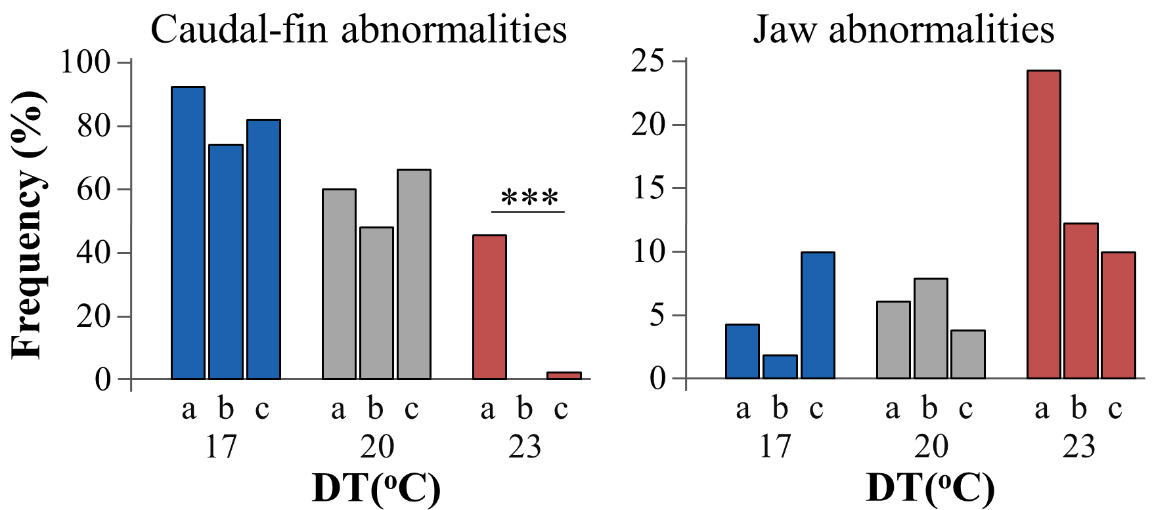


**Fig. S2**: Frequency of the caudal-fin and jaw abnormalities in the replicates (a, b, c) of the different thermal treatments. ***, p<0.001(G-test).
